# Comprehensive Analysis of Metabolome and Transcriptome in Fruits and Roots of Kiwifruit

**DOI:** 10.3390/ijms24021299

**Published:** 2023-01-09

**Authors:** Long Zhang, Zhengmin Tang, Hao Zheng, Caihong Zhong, Qiong Zhang

**Affiliations:** 1 Key Laboratory of Plant Germplasm Enhancement and Specialty Agriculture, Wuhan Botanical Garden, Chinese Academy of Sciences, Wuhan 430074, China; 2 Engineering Laboratory for Kiwifruit Industrial Technology, Chinese Academy of Sciences, Wuhan 430074, China

**Keywords:** kiwifruit, metabolome, transcriptome, dihydrochalcone, flavonol

## Abstract

Kiwifruit (*Actinidia chinensis*) roots instead of fruits are widely used as Chinese medicine, but the functional metabolites remain unclear. In this study, we conducted comparative metabolome analysis between root and fruit in kiwifruit. A total of 410 metabolites were identified in the fruit and root tissues, and of them, 135 metabolites were annotated according to the Kyoto Encyclopaedia of Genes and Genomes (KEGG) pathway. Moreover, 54 differentially expressed metabolites (DEMs) were shared in root and fruit, with 17 DEMs involved in the flavonoid pathway. Of the 17 DEMs, three flavonols (kaempferol-3-rhamnoside, L-Epicatechin and trifolin) and one dihydrochalcone (phloretin) showed the highest differences in the content level, suggesting that flavonols and dihydrochalcones may act as functional components in kiwifruit root. Transcriptome analysis revealed that genes related to flavonols and dihydrochalcones were highly expressed in root. Moreover, two AP2 transcription factors (TFs), *AcRAP2-4* and *AcAP2-4*, were highly expressed in root, while one bHLH TF *AcbHLH62* showed extremely low expression in root. The expression profiles of these TFs were similar to those of the genes related to flavonols and dihydrochalcones, suggesting they are key candidate genes controlling the flavonoid accumulation in kiwifruit. Our results provided an insight into the functional metabolites and their regulatory mechanism in kiwifruit root.

## 1. Introduction

Kiwifruit (*Actinidia chinensis*) is native to China, and there are 54 species and about 75 taxa in the genus *Actinidia* [1]. Although the kiwifruit industry initially focused on the green-fleshed variety ‘Hayward’ (*A. chinensis* var. *deliciosa*), it later developed several varieties of *A. chinensis*, *Actinidia arguta* (*A. arguta*), *Actinidia eriantha* (*A. eriantha*), and interspecific hybrids, thus increasing the availability of green, yellow, and red flesh varieties [2]. ‘Donghong’ kiwifruit (*A. chinensis*) is popular among consumers worldwide due to its rich aroma and bright red flesh. Additionally, the ready-to-eat kiwiberry (*A. arguta*) and *A. eriantha* have also become popular in China. The fruit of *A. arguta* is smaller than a fuzzy kiwifruit and has thin, smooth, and edible skin [3]. *Actinidia eriantha* has an extremely high content of vitamin C (VC). The fruit is densely covered with white tomentum, and the fruit pulp is dark green [4]. The yellow-fleshed hybrid ‘Jinyan’ (*A. eriantha* × *A. chinensis*) is popular around the world owing to its taste and excellent shelf life [5].

The kiwifruit is extremely nutritious and rich in metabolites with potential healthcare effects and medicinal values [6,7,8,9,10,11]. The roots of kiwifruit contain triterpenoids and flavonoids, which have pharmacological effects, such as anti-tumor, anti-inflammatory, and anti-viral [12,13]. The root extracts of kiwifruit might be used as immunopotentiating reagents and can affect the proliferation of cancer cells [14,15]. FOLFOX4 has been applied widely as the first-line drug of gastric cancer chemotherapy, and FOLFOX4 combined with kiwifruit root was more effective with a high clinical remission rate, mild adverse effects and significantly longer survival time [16]. Thus, the root of kiwifruit was usually used in medicine. Besides the root of kiwifruit, the fruit is also rich in metabolites. The juice of kiwifruit can help patients with type 2 diabetes mellitus as it has anti-inflammatory and antioxidant effects [17]. Kiwifruit can also relieve constipation by promoting gastrointestinal motility and improving blood pressure [18,19,20,21]. The flesh color is associated with specific types of metabolites in different kiwifruit varieties. For example, ‘Jinshi 1’ (*A. chinensis*) contains high levels of β-carotene, lutein, zeaxanthin, β-cryptoxanthin, and other substances that make the flesh turn yellow [22]. The flesh of ‘Ganlv 1’ (*A. eriantha*) and ‘Yongfengyihao’ (*A. arguta*) is rich in chlorophyll, which causes the flesh to appear green [23,24]. The flesh of ‘Hongyang’ (*A. chinensis*) is red due to high levels of anthocyanins [25]. Besides the metabolites of pigments, there are other metabolites associated with health in the flesh of the kiwifruit, including vitamins, folate, phenolic compounds, minerals, and dietary fiber [26,27]. These substances in different kiwifruit varieties might affect the taste of the fruit due to the differences in their content [28].

In flavonoid biosynthesis pathway, phenylalanine is catalyzed to form coumaroyl-CoA under the action of phenylalanine ammonia-lyase (PAL), cinnamate-4-hydroxylase (C4H), 4-coumarate-CoA ligase (4CL), and other enzymes. Coumaroyl-CoA is converted to chalcone under the action of chalcone synthase (CHS), and chalcone is catalyzed by chalcone isomerase (CHI) to produce flavanone. Flavanone generates dihydroflavonol under the catalysis of flavanone-3-hydroxylase (F3H), flavonoid-3’-hydroxylase (F3’H), and flavonoid-3’,5’-hydroxylase (F3’5’H). Dihydroflavonol generates leucocyanidin under the catalysis of dihydroflavonol 4-reductase (DFR). Finally, anthocyanin is generated under the action of anthocyanidin synthase (ANS) and anthocyanidin reductase (ANR). Besides the above structural genes, some key regulatory factors, such as MYB and bHLH, are also closely related to anthocyanin synthesis [29].

Although some studies have investigated the function of some metabolites in kiwifruit, few have elucidated the differences in the metabolites between the root and fruit among different representative varieties. To determine the metabolite differences between the roots and flesh of different kiwifruit varieties, the root of ‘Donghong’ (DH) and fruits of DH, ‘Huate’ (HT), ‘LD133’ (RZ) and ‘Jinyan’ (JY) were selected for study. We combined metabolomic and transcriptomic data to determine major metabolic and gene expression profiles and examine the differences in functional compounds between fruit and root. In this study, we provided a profile of metabolites and regulatory mechanism in kiwifruit, which might be used for breeding and comprehensive application.

## 2. Results

### 2.1. Difference in Flesh Color among Four Kiwifruit Varieties

**Figure 1:**
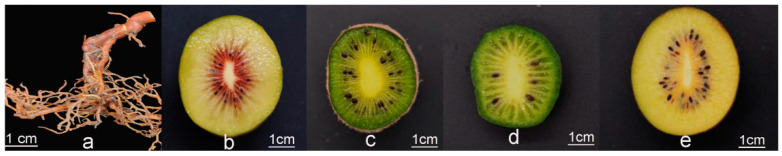
The morphological characteristics of the root and fruit of kiwifruit varieties used in this study. (**a**) root of ‘Donghong’ (R); (**b**) fruit of ‘Donghong’ (DH); (**c**) fruit of ‘Huate’ (HT); (**d**) fruit of ‘LD133’ (RZ); (**e**) fruit of ‘Jinyan’ (JY).

Kiwifruit plants have high morphological variation and possess green, red, or yellow flesh. To establish the metabolic and gene expression profiles between root and fruit of different kiwifruit species, we collected the root of ‘Donghong’ (R) and four typical varieties, ‘Huate’ (HT), ‘LD133’ (RZ), ‘Donghong’ (DH), and ‘Jinyan’ (JY), consisting of the species *A. eriantha, A. arguta*, and *A. chinensis* and an interspecies hybrid of *A. eriantha* and *A. chinensis*. The flesh of HT and RZ is green, the flesh of DH is red, and the flesh of JY is yellow (Figure 1). Generally, kiwifruit varieties were mostly propagated by grafting, the roots of DH were derived from tissue culture seedlings of DH, and plants had been transplanted in orchards for 5 years.

### 2.2. The Metabolome Profiling of Fruit and Root in Kiwifruit

**Figure 2:**
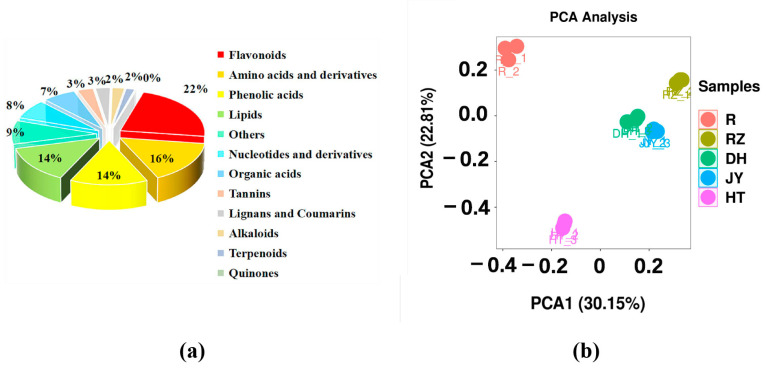
Metabolome profiling of kiwifruit flesh and root. (**a**) The classification of total metabolites in root and flesh samples; (**b**) PCA of metabolites in root and flesh samples.

In total, 410 annotated specialized metabolites were identified in at least one sample. The analysis of the metabolites was mainly divided into the positive ion mode (POS) and the negative ion mode (NEG). The results showed that 201 and 209 metabolites were detected in the POS and NEG modes, respectively, of which 73 and 62 metabolites were annotated to the KEGG pathway (Table 1). Among these metabolites, 92 flavonoids, 58 phenolic acids, 57 lipids, 65 amino acids and their derivatives, 31 nucleotides and their derivatives, 27 organic acids, 13 tannins, 12 lignans and coumarins, 10 alkaloids, seven terpenoids, and one quinone were quantified in the kiwifruit flesh and root. The flavonoids accounted for approximately 22% of all metabolites, followed by amino acids and their derivatives (16%), phenolic acids (14%), and lipids (14%) (Figure 2a). In the ‘Donghong’ root, 375 metabolites were detected, of which 84 metabolites were flavonoids, accounting for the largest proportion (22%), followed by amino acids and their derivatives (59.16%), phenolic acids (53.14%), lipids (52.14%), nucleotides and their derivatives (29.8%), and organic acids (26.7%). The principal component analysis (PCA) was performed with all samples based on metabolic signals. The results of the PCA revealed that components 1 and 2 explained 30.15% and 22.81% of variability, respectively (Figure 2b). Components 1 and 2 separated the root and flesh samples, indicating significant metabolic diversity between them. Further observations revealed that the flesh of DH, JY, and RZ was closer to each other but far from the flesh of HT in the PCA diagram, indicating that DH, JY, and RZ have more similar metabolic profiles in fruits.

### 2.3. Identification of Differentially Expressed Metabolites (DEMs)

**Figure 3:**
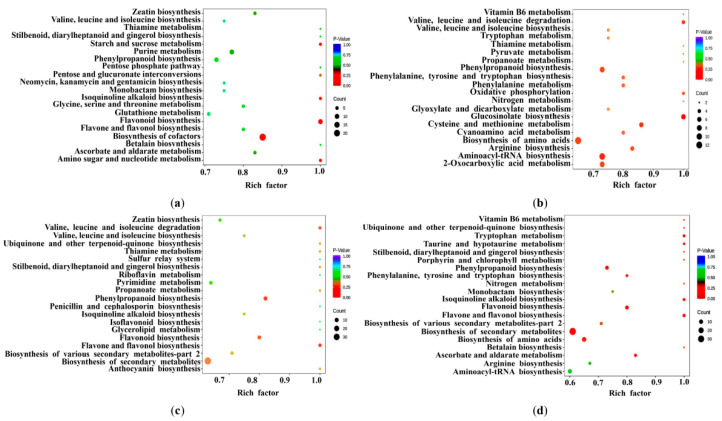
The KEGG enrichment analysis of the DEMs. (**a**) DH vs. R; (**b**) DH vs. JY; (**c**) DH vs. HT; (**d**) DH vs. RZ.

To better understand the metabolic variation in the flesh and root, we compared the similarities and differences in metabolites and found that 272 metabolites were shared in all flesh and root samples. Of the 272 metabolites, 135 metabolites were annotated to the KEGG pathway. We performed a KEGG enrichment analysis of with 135 metabolites in four comparison groups (Figure 3). There were 380 metabolites detected in DH roots and 371 metabolites detected in DH fruits. In the comparison between roots and fruits of DH, 275 differential metabolites were detected, of which 96 DEMs were annotated to the KEGG pathway, with 50 and 46 metabolites in the POS and NEG modes, respectively. In 96 DEMs, amino acids and nucleic acids with derivatives accounted for 38%, followed by flavonoids (14%), organic acids (13%) and phenolic acids (9%). The phloretin was the most abundant in the DH roots, while cyanidin 3-O-glucoside was significantly enriched in the DH fruits, both of them belonging to flavonoids.

In addition, 370, 315 and 377 metabolites were detected in JY, HT and RZ fruits, respectively. A comparison between the flesh of DH and the flesh of JY, HT, and RZ showed that 65, 79 and 76 DEMs were annotated to the KEGG pathway, and the DEMs were significantly enriched in the phenylpropanoid metabolism, flavonoid biosynthesis and biosynthesis of secondary metablites, respectively (Figure 3b–d). The DEMs annotated to the KEGG pathway were summarized, and the heat map of the shared DEMs was constructed.

**Figure 4:**
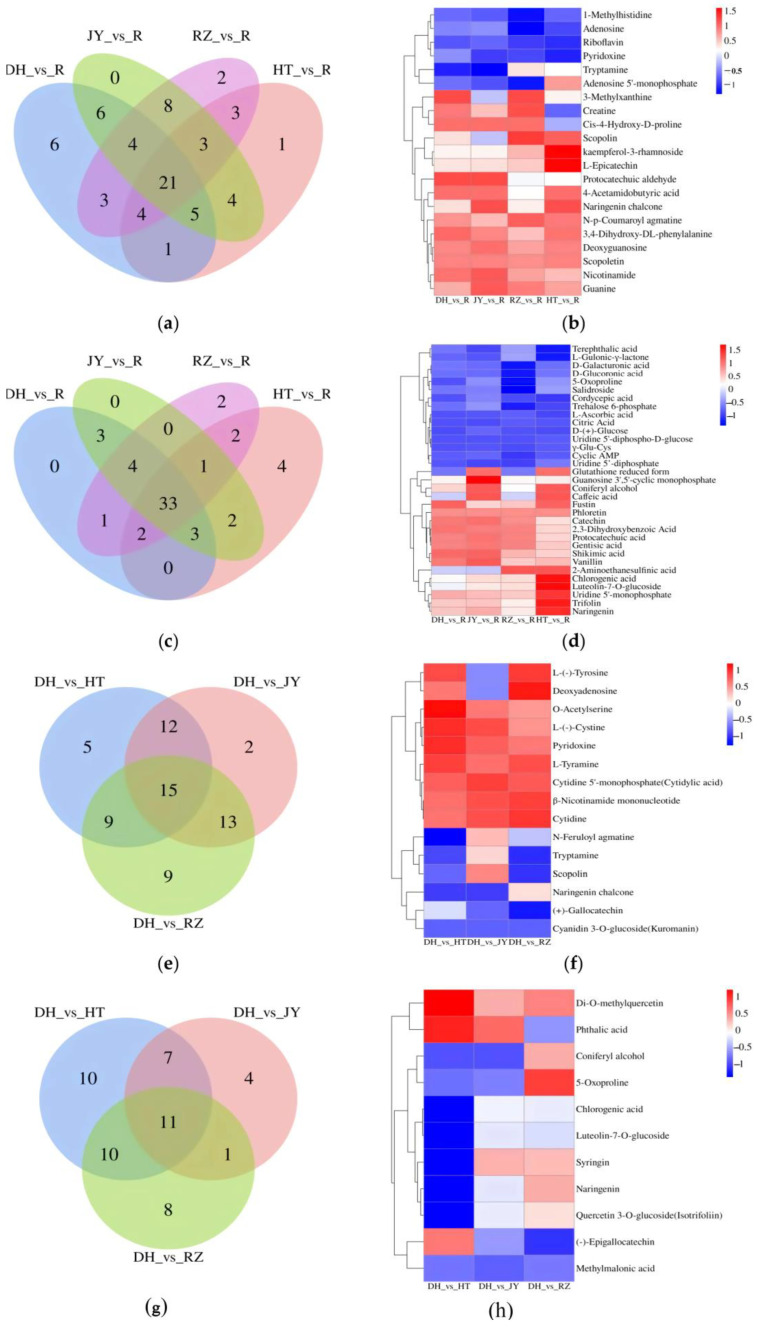
Venn diagrams and heatmaps of DEMs. (**a**,**e**) The Venn diagrams of DEMs in the POS mode; (**b**,**f**) the heatmaps of shared DEMs in the POS mode; (**c**,**g**) the Venn diagrams of DEMs in the NEG mode; (**d**,**h**) the heatmaps of shared DEMs in the NEG mode.

**Figure 5:**
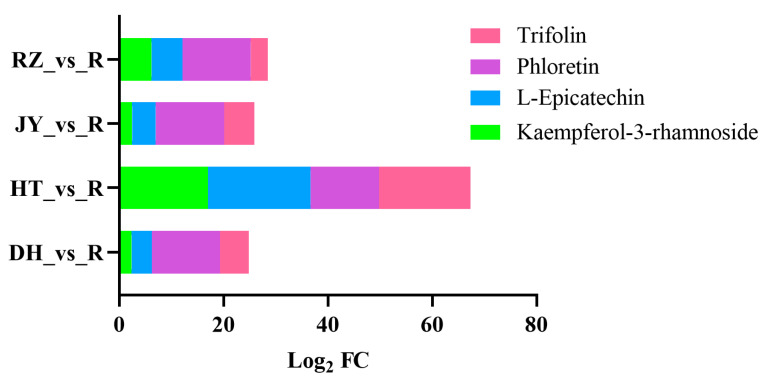
Metabolites that were significantly different between the root and flesh.

A comparison between the root and four fruit samples showed 128 DEMs were identified between the root and fruit, including 96 DEMs between root and fruit of DH. Of these DEMs, 54 DEMs were detected not only between the root and fruit of DH, but also shared between the root and each fruit sample. There were 21 and 33 DEMs in the POS and NEG modes, respectively (Figure 4a–d). Among the 54 DEMs, there were 17 in the flavonoid pathway. We further increased the screening threshold (|Log_2_FC| ≥ 2), and three flavonols (kaempferol-3-rhamnoside, L-Epicatechin and trifolin) and one dihydrochalcone (phloretin) were identified not only between the root and fruit of DH, but also between the root and fruit of three cultivars (Table 2 and Figure 5). Kaempferol-3-rhammoside, L-Epicatechin and trifolin were bioactive compounds classed as flavonols, and phloretin belonged to a class of dihydrochalcones. The three flavonols and phlotetin showed highest differences in the content level between roots and fruits of different cultivars, suggesting that they were highly expressed in kiwifruit roots.

A comparison between the flesh of DH and the flesh of JY, HT, and RZ showed that there were 15 and 11 shared DEMs under the POS and NEG modes, respectively (Figure 4e–h). The contents of cyanidin-3-O glucoside, chlorogenic acid, and luteolin-7-O-glucoside in the flesh of DH were higher than those in the flesh of the other three varieties of kiwifruit. These three substances are involved in the biosynthesis pathway of flavonoids [30]. In comparison between RZ and JY fruits, 226 DEMs were identified, 81 of which were annotated in KEGG pathway and were mainly enriched in the biosynthesis of secondary metabolites. In a comparison of HT and RZ fruits found 286 DEMs, 89 DEMs were annotated in the KEGG pathway, and in the comparison of JY and HT fruits detected 270 DEMs, 81 of them were annotated in the KEGG pathway, which were highly enriched in the flavonoid biosynthesis pathway.

### 2.4. Identification of Differentially Expressed Genes (DEGs)

**Figure 6:**
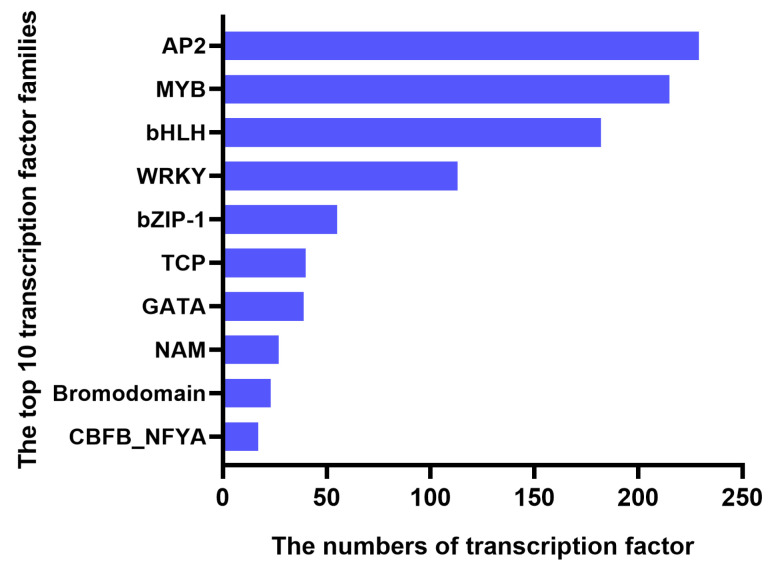
The number of transcription factors involved in the top 10 transcription factor families. The *X*-axis represents the number of transcription factors, and the *Y*-axis represents the top 10 transcription factor families.

In total, five samples, each with three replicates, were transcriptome-sequenced, and 32,940 DEGs were detected in R, DH, HT, RZ, and JY (Table 3). Moreover, 1065 TFs were identified and classified into 34 TF families (Figure 6), the majority of which were “AP2”, “MYB”, “bHLH”, and “WRKY”.

**Figure 7:**
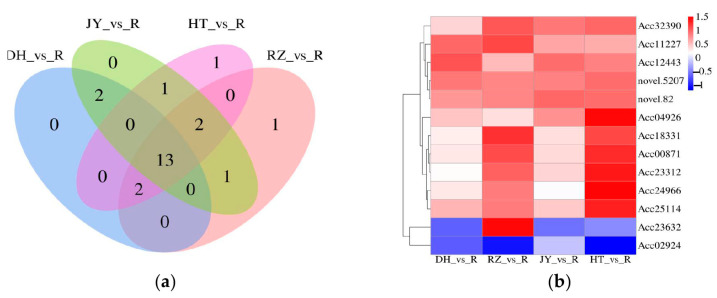
Venn diagram and heatmap of DEGs. (**a**) The Venn diagram of DEGs in the four comparison groups. (**b**) The heat map of the shared DEGs in the four comparison groups. Taking A_vs_B as an example, A is regarded as the control group, and B is the experimental group.

Among the four comparison groups of roots and fruits, the DEGs involved in flavonoid biosynthesis were summarized, and a Venn diagram was constructed. There were 13 shared DEGs (Figure 7a), and the heat map of the shared DEGs in the four comparison groups was constructed (Figure 7b).

### 2.5. Functional Classification of DEGs

The transcriptome data were analyzed, and the functional description of the DEGs was summarized (Table 4), where *Acc24966*, *Acc18331*, *novel.82*, and *Acc23312* were found to encode chalcone synthase, flavonoid-3’-hydroxylase, trans-cinnamate-4-monooxygenase, and caffeoyl-CoA-O-methyltransferase, respectively. They were key enzymes involved in the flavonoid biosynthesis pathway. The *Acc04926* and *Acc25114* were found to be AP2 TFs; they can specifically bind to a GCC/box, which is associated with plant resistance. The results of evolutionary tree analysis showed that *Acc04926* and *Acc25114* were homologous with CsRAP2-4 like and ArERF4-like, and they were called *AcRAP2-4* and *AcAP2-4,* respectively (Figure S1). The *Acc02924* (called *AcbHLH62*) is a bHLH TF (Figure S2), which forms a protein complex with MYB and WD40 (as MYB-bHLH-WD40) to regulate the promoter of structural genes in the anthocyanin biosynthesis pathway. It can upregulate structural gene expression and promote anthocyanin biosynthesis and accumulation.

### 2.6. Analysis of Association between DEMs and DEGs

Generally, metabolites are associated with genes. The metabolite group represents the most downstream stage in the whole dynamic system. Within the gene–protein–metabolite–phenotype framework, metabolites can regulate physiological processes and phenotypes. Therefore, Pearson’s correlation coefficients (PCC) between four DEMs and 13 DEGs were calculated, and the results showed that *Acc24966*, *Acc18331, novel.82, Acc23312, Acc04926* (*AcRAP2-4*)*, Acc25114 (AcAP2-4*), and *Acc02924 (AcbHLH62*) were strongly correlated with the four candidate metabolites (Table 5).

### 2.7. Validation of Genes Related to Flavonoid Biosynthesis Using RT-qPCR

**Figure 8:**
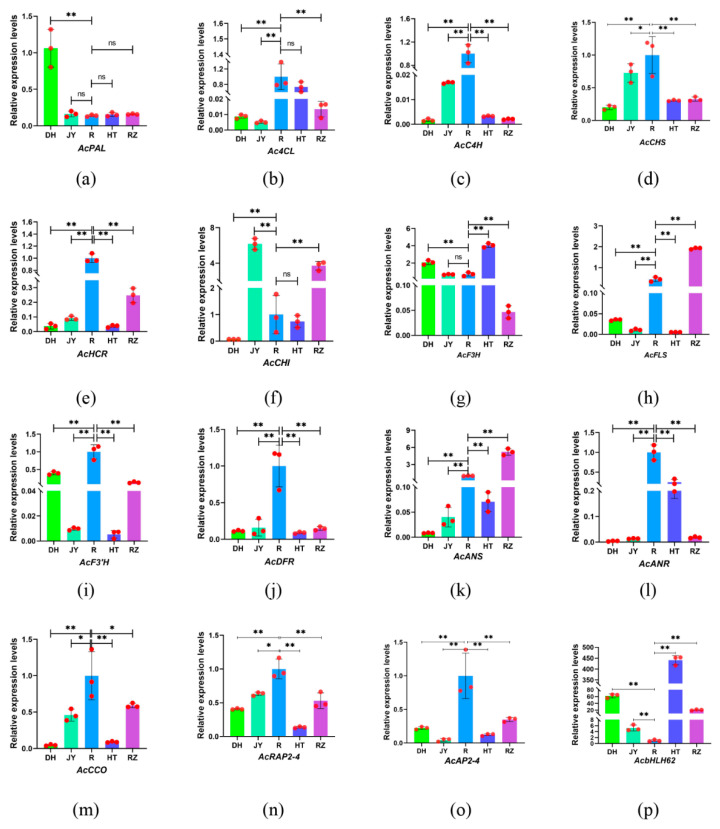
Relative expression of structural genes and transcription factors in the roots and four fruit samples. (**a**) *AcPAL*; (**b**) *Ac4CL*; (**c**) *AcC4H*; (**d**) *AcCHS*; (**e**) *AcHCR*; (**f**) *AcCHI*; (**g**) *AcF3H*; (**h**) *AcFLS*; (**i**) *AcF3′H*; (**j**) *AcDFR*; (**k**) *AcANS*; (**l**) *AcANR*; (**m**) *AcCCO*; (**n**) *AcRAP2-4*; (**o**) *AcAP2–4*; (**p**) *AcbHLH62*. The results of the independent samples *t*-test showed that there was a significant difference between the groups; * indicates *p* < 0.05 and ** indicates *p* < 0.01.

In the biosynthesis pathway of flavonoids, structural genes and regulatory factors, such as CHS, C4H, MYB, bHLH, play a crucial role. Therefore, four structural genes and three genes of transcription factors related to flavonoid biosynthesis were identified and amplified by RT-qPCR. The results showed that the expression patterns of eight structural genes, including *Ac4CL, AcC4H*, *AcCHS*, *AcHCR, AcDFR, AcANR, AcCCO*, and *AcF3′H*, were similar to those of *AcRAP2-4* and *AcAP2–4,* which was expressed significantly more in the roots than in the fruits. In contrast, *AcbHLH62* showed the lowest expression in the roots (Figure 8).

### 2.8. Genes Related to Biosynthesis of Dihydrochalcones and Flavonols Were Activated in Kiwifruit Roots

**Figure 9:**
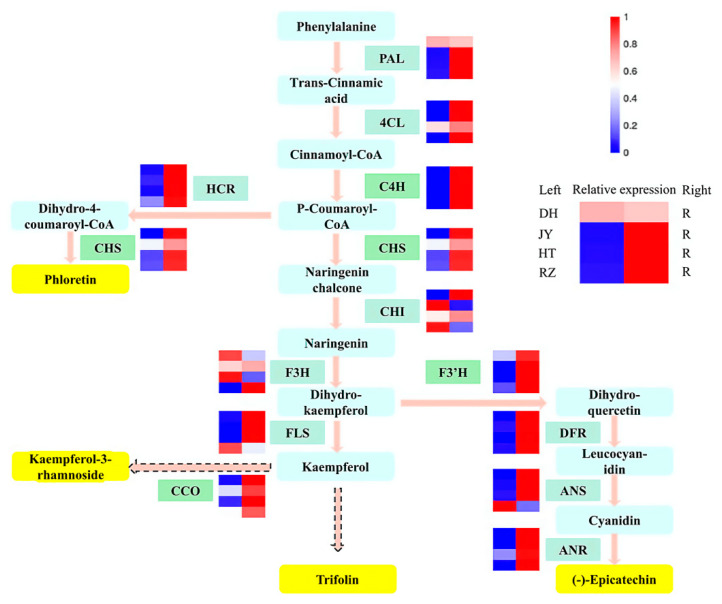
The regulatory network of flavonoid biosynthesis. The color scale from blue to bright red indicates relative expression levels. The compounds in the yellow round-angle boxes are the upregulated DEMs; the genes in the green rectangular boxes are DEGs. Solid arrows indicate only one reaction step, and dashed arrows indicate multiple reaction steps. PAL: phenylalanine ammonia-lyase; 4CL: 4-coumarate-CoA ligase; C4H: cinnamate-4-hydroxylase; HCR: hydroxycinnamoyl-CoA reductase; CHS: chalcone synthase; CHI: chalcone isomerase; F3H: flavanone-3-hydroxylase; FLS: flavonol synthase; CCO: caffeoyl-CoA-O-methyltransferase; F3′H: flavonoid-3’-hydroxylase; DFR: dihydroflavonol 4-reductase; ANS: anthocyanidin synthase; ANR: anthocyanidin reductase.

To better understand the relationship between metabolites and genes in the flavonoid biosynthesis pathway, all results of metabolites and genes were combined to establish a network and display the relationship between gene expression and metabolite accumulation more intuitively (Figure 9). Among 14 structural genes, *Ac4CL*, *AcC4H, AcCHS, AcHCR, AcF3′H, AcDFR, AcANR*, and *AcCCO* showed a higher expression in the roots than in the four fruit samples. The four metabolites, including kaempferol-3-rhamnoside, L-Epicatechin, phloretin, and trifolin, accumulated more in the roots. Transcription factors regulate gene expression by binding to the promoter regions of all genes. Therefore, we speculated that the AP2 and bHLH transcription factors can regulate the expression of *AcC4H, AcCHS, AcF3′H*, and *AcCCO* in the flavonoid biosynthesis pathway and lead to the differential accumulation of metabolites in the roots and fruits.

## 3. Discussion

### 3.1. DEMs between Kiwifruit Roots and Fruits Were Enriched in Dihydrochalcones and Flavonols

Kiwifruit is highly nutritious and has potential medicinal properties [31]. Metabolic profiles can help to distinguish different species and determine their nutritional characteristics [32]. Previous studies on kiwifruit metabolites have mostly focused on the flesh of the fruit [33,34,35]. In this study, we conducted a metabolomic analysis on the roots and different varieties of red, green, and yellow kiwifruit flesh. The results of the PCA analysis showed that the root can be separated from fruit samples, based on the differences in the metabolites. Kiwifruit roots contain polysaccharides and glycosides, terpenoids, tannins, sterols, flavonoids and other chemical components, which have usually been used in traditional Chinese medicine [36]. We focused on identifying differential metabolites between roots and flesh. In total, four metabolites, including kaempferol-3-rhamnoside, L-epicatechin, phloretin and trifolin, were selected as candidates based on a metabolome analysis. Kaempferol-3-rhammoside, L-Epicatechin and trifolin were bioactive compounds classed as flavonols, and phloretin belonged to a class of dihydrochalcones. Flavonols play a crucial role in plant growth, attract pollinators, protect the plant from UV light, insects, fungi and viruses, and act as hormone regulators [37]. Due to a lack of flavonols, mutants of maize and petunia failed to germinate, but the addition of kaempferol at pollination time compensated for this deficiency and the mutants were still able to grow and develop normally [38]. In addition, flavonols can not only scavenge free radicals and have antioxidant effects, but also detoxify and have anti-inflammatory effects, improve human immunity, enhance cardiovascular and cerebrovascular blood flow, and block platelet agglutination [39]. Besides antioxidant effects as other flavonoids, dihydrochalcone compounds have physiological and pharmacological activities [40]. Phloretin has an inhibitory effect on SGLT-2, making it therapeutic for diabetes [41]. In addition, phloretin has antioxidant and anti-aging properties, it can block tumor cell growth by blocking cell cycle proteins and cell cycle protein-dependent kinases, and induce apoptosis by activating mitochondria-mediated cell death [42,43]. In this study, we identified four differential metabolites, which were highly rich in roots of kiwifruit. Phloretin can block tumor cell growth and induce apoptosis, and flavonols were beneficial for cardiovascular and cerebrovascular diseases. Therefore, flavonols and phloretin may be crucial components in the root of kiwifruit in traditional Chinese medicine.

### 3.2. Metabolome Profiling Is an Efficient Approach to Estimate the Genetic Relationship between Cultivars

Due to the accumulation of anthocyanins, such as cyanidin-3-O glucoside, the flesh of the fruit of DH appeared to be a red color. In this study, we found that the content of cyanidin-3-O glucoside in the flesh of DH was significantly higher than that in the flesh of JY, HT, and RZ. It was reported that the cyanidin-3-O glucoside was enriched in red-fleshed kiwifruit in a previous study [44]. Furthermore, the contents of chlorogenic acid and luteolin-7-O-glucoside in the flesh of DH were also higher than those in the flesh of the other three varieties, and these two substances were also involved in the biosynthesis pathway of flavonoids. Based on the flavonoid biosynthesis pathway, chlorogenic acid and luteolin-7-O-glucoside can be used as precursors for the synthesis of cyanidin-3-O glucoside. In addition, the DH and JY were clustered together, and separated from RZ and HT in the PCA analysis. This conclusion confirmed the results of another study, where the JY was a hybrid of *A. eriantha* as the female parent and *A. chinensis* as the male parent, whose shape, hair content, skin, and flesh were of a similar color to those of the male parent [5]. Metabolome profiling could be used as an efficient approach to estimate the genetic relationship between cultivars.

### 3.3. The Biosynthesis of Dihydrochalcones and Flavonols in Kiwifruit Roots Is Regulated at the Transcriptional Level

The structural and regulatory genes involved in flavonoid biosynthesis have been identified and studied in many plants [45,46]. Advancements in molecular biology techniques have increased the understanding of the mechanism of gene regulation and the level of gene expression. Therefore, metabolomics and transcriptomics are extensively used together to analyze phenotypic traits [47]. In this study, metabolomic data were analyzed along with the transcriptome to identify genes involved in flavonoid biosynthesis. The results facilitated exploring differences in metabolites in the root and flesh. We identified four candidate structural genes (*AcC4H*, *AcCHS, AcF3′H,* and *AcCCO*) and three genes of transcription factors (*AcRAP2-4*, *AcAP2–4* and *AcbHLH62*). The results of the RT-qPCR analysis showed that *AcC4H*, *AcCHS*, *AcF3′H, AcCCO, AcRAP2-4* and *AcAP2-4* had a significantly higher expression in the roots than in the fruits. The transcription factor AP2 belongs to a family of plant-specific transcription factors that are found extensively in plants and are involved in the signal transduction of various physiological and biochemical responses, such as flower organ formation, disease resistance, stress resistance, and hormone response [48]. The bHLH transcription factors regulate eukaryotic growth and development and are essential for plant growth and development. They are also involved in various types of stress in plants [49]. We speculated that the transcription factors AP2 and bHLH bind to the promoter regions of structural genes and regulate their expression, thus leading to differences in the metabolites between roots and fruits. Based on these results, we established a regulatory network for flavonoid biosynthesis, which showed the role of the genes in the pathway more intuitively.

By combining the results of the metabolome and transcriptome analysis, we determined the differences in the metabolites between the roots and fruits of different kiwifruit varieties, and identified candidate genes and TFs related to functional metabolites in the flavonoid biosynthesis pathway. The differences in the metabolite profiles between roots and fruits and the variation among kiwifruit varieties facilitate the application of kiwifruit in the field of pharmacy and processing.

## 4. Materials and Methods

### 4.1. Plant Materials

Mature kiwifruits of four cultivars (i.e., ‘Donghong’, ‘Jinyan’, ‘White’, and ‘LD133’) and the roots of ‘Donghong’ grown in the same environment were collected from the National Germplasm Repository of Kiwifruit in the Wuhan Botanical Garden, Chinese Academy of Sciences. All plants used in the experiment started bearing fruits more than five years before the study was conducted. Decreased fruit growth (size and weight), close to maximum dry matter, and 100% black seeds are considered to be the first few signs of mature kiwifruit [50]. The fruits were randomly selected from each plant at a mature stage with a maximum of dry matter. One replicate consisted of 10 fruits, and each group of samples had three biological replicates. The roots of DH cultivar come from 15 individuals, and the main root were selected from 10–12 cm below ground in the mature fruit stage, then cleaned and divided into three parts for biological replicates. The fruits were peeled, and all fruit and root samples were cut into small chunks, frozen immediately in liquid nitrogen, and stored at −80 °C until further use.

### 4.2. Metabolite Extraction and Parameter Setting

The freeze-dried fruits and roots were pulverized using a mixer mill (Retsch, Bavaria, Germany) with a zirconia bead for 1.5 min at 30 Hz. Sample powders (100 mg) were weighed and extracted overnight at 4 °C with 0.6 mL of 70% aqueous methanol (Dingshengxing, Tianjin, China). The extracts were centrifuged at 10,000 *g* for 10 min (Sigma, Beijing, China). The supernatant was removed by solid phase extraction (SPE) cartridges (CNWBOND Carbon-GCB SPE Cartridge; 250 mg, 3 mL; ANPEL, Shanghai, China), and filters (SCAA-104; 0.22 µm pore size; ANPEL, Shanghai, China) before they were injected for performing ultra-performance liquid chromatography-tandem mass spectrometry (UPLC-MS/MS, Shimadzu, Beijing, China) analysis.

An ultra-performance liquid chromatography-electro-spray ionization-tandem mass spectrometry (UPLC-ESI-MS/MS) system (UPLC, Shim-pack UFLC SHIMADZU CBM30A system, Shimadzu, Beijing, China; MS, Applied Biosystems 4500 Q TRAP, Shimadzu, Beijing, China) was used to analyze kiwifruit extracts. A UPLC column (Waters ACQUITY UPLC HSS T3 C18 (1.8 µm × 2.1 mm × 100 mm) was used for the chromatographic separation of the metabolites (Waters, Beijing, China). The mobile phase was solvent A (pure water with 0.04% acetic acid) and solvent B (acetonitrile with 0.04% acetic acid) (Sigma-aldrich, Shanghai, China). The samples were measured using a gradient program with the starting conditions of 95% A and 5% B. A linear gradient was followed to change the composition to 5% A and 95% B within 10 min, and this composition was maintained for 1 min. Then, the composition was changed to 95% A and 5% B within 0.1 min and maintained for 2.9 min. The temperature of the column oven (Thermo fisher, Suzhou, China) was maintained at 40 °C, and the volume of the sample injected was 4 µL (Eppendorf, Beijing, China).

The triple quadrupole linear ion trap mass spectrometer (Applied Biosystems, Beijing, China) was operated in both positive and negative ion modes and equipped with an ESI turbo ion-spray interface. The operation parameters of the ESI source were as follows: the ion source was a turbo spray; the source temperature was 550 °C; the ion spray voltage (IS) ranged from 5500 V to 4500 V; ion source gas I, gas II, and curtain gas were set at 50, 60, and 30 pounds per square inch (PSI), respectively; the collision gas was high. Targeted MS/MS scans were acquired in the multiple reaction monitoring (MRM) mode, and each ion pair was scanned and detected using optimised declustering potential (DP) and collision energy (CE).

### 4.3. RNA Extraction and Sequencing

The four fruit samples and one root sample were used for RNA sequencing, and each sample included three biological replicates. We performed RNA extraction, detection, construction of the cDNA library, and sequencing, as described in the Ref. [35]. Details of RNA extraction are as follows: samples of plant tissues were rapidly ground in liquid nitrogen, 50–150 mg of each sample and 1 mL of TRNzol-A used with 0.2 mL of chloroform was shaken vigorously for 15 s and left at room temperature for 3 min. The tubes were centrifuged at 4 °C, 12,000 rpm for 10–15 min, and divided into three layers, the RNA is mainly in upper aqueous phase. The aqueous phase (about 500 uL) of samples were transfered to new tubes, and added an equal volume of isopropanol, then mixed well and leaved for 20–30 min at room temperature. The tubes were centrifuged at 4 °C, 12,000 rpm for 10 min, and the supernatant was removed. A total of 1 mL of 75% ethanol (Sigma-aldrich, Shanghai, China) was added to wash the precipitate twice. The precipitate was centrifuged at 4 °C, 5000 rpm for 3 min, and dried at room temperature. Finally, 30 uL RNase-free ddH_2_O was added to fully dissolve the RNA. The purity and integrity of the RNA samples were determined using NanoPhotometer spectrophotometer (IMPLEN; Westlake Village, CA, USA) and Agilent 2100 Bioanalyzer (Agilent Technologies; Santa Clara, CA, USA), respectively. The sequencing library was constructed using the NEBNext^®^ Ultra™ RNA Library Prep Kit for Illumina^®^. After constructing the library, initial quantification was conducted using a Qubit 2.0 Fluorometer (Life Technologies; Carlsbad, CA, USA). The library was diluted to 1.5 ng/µL, and the insert size of the library was evaluated using the Agilent 2100 Bioanalyzer (Agilent, Shanghai, China) to ensure the quality of the library. Then, the different libraries were pooled according to the effective concentration, and sequenced on the Illumina platform. Clean RNA-Seq data were deposited in Sequence Read Archive (SRA) at the National Center for Biotechnology Information (NCBI) with the accession number PRJNA916812.

### 4.4. Analysis of TFs and DEGs

The TFs of the plants were identified using the plant TF database PlantTFDB 4.0 (http://planttfdb.gao-lab.org/, 1 January 2020). A total of 165 plant species in the database were used as a reference for Ensembl gene IDs, the TF was screened directly. For genes that did not have Ensembl gene IDs, SUPERFAMILY and Pfam annotations were performed using the InterPro Scan software to obtain the SUPERFAMILY and Pfam IDs of each gene. The DBD database (transcription factor prediction database) was then used to predict the corresponding TFs for each SUPERFAMILY and Pfam that was annotated.

The adaptor sequences and low-quality sequence reads were removed from the data sets to obtain clean reads which were then mapped to Red5 kiwifruit reference genome (https://www.ncbi.nlm.nih.gov/nuccore/CM009676, accessed on 30 March 2018). To avoid the effects of sequencing depth and gene length, fragments per kilobase of transcript per million fragments mapped (FPKM) usually was used to reflect gene expression from RNA-seq [51]. Quantification of gene expression levels was estimated by FPKM. Differential expression analysis in root and fruits of 4 cultivars were performed using DESeq2. A negative binomial distribution model was used for calculating the *p*-value, and padj was introduced to correct the *p*-value for hypothesis testing and controlling the proportion of false positives [52]. The differential gene screening standard was |log2(FoldChange)| > 1 and padj < 0.05.

### 4.5. Real-Time Quantitative PCR (RT-qPCR)

The RNA sample was extracted from ripening kiwifruit roots and flesh using the Total Plant RNA Microextraction Kit (Magen, Beijing, China). Agarose gel electrophoresis and ultra-micro nucleic acid protein detector EVA3100 (Monad Biotech Co., Ltd, Zhuhai, China.) were used to determine the quality and concentration of the RNA samples, respectively. The extracted RNA was used as a template and reverse-transcribed to cDNA using the SYBR^®^ Premix Ex Taq™ II (TAKARA, Dalian, China) kit, and the entire operation was conducted on ice. The primer sequences for the gene were synthesized by Tsingke Biotechnology Co., Ltd. (see Supplementary Material Table S1). The RT-PCR analysis was performed using the TB Green Premix Ex Taq II kit (Takara, Dalian, China) with the reverse-transcribed cDNA as a template. The entire procedure was performed on ice and in the dark. The reaction system was as follows: in a 384-well plate, 9 µL of premix was added to each well. The volume of TB Green Premix Ex Taq II used was 5 µL, and the volume of Forward and Reverse primers used was 1 µL each. The volume of ROX Reference Dye II used was 1 µL, and the volume of RNA-free water used was 1 µL. Finally, 1 µL of cDNA was added (diluted to 500 ng/µL). Gene expression was measured using a 384-well QuantStudio 6 Flex real-time fluorescence PCR assay system (Thermo fisher, Beijing, China). The conditions used for the PCR analysis were as follows: 95 °C for 30 s, followed by 40 cycles of 95 °C for 3 s, 60 °C for 30 s, and 72 °C for 20 s; the total reaction volume was 10 µL. At the end of the experiment, the melting curve was analyzed using the default parameters. The *β-ACTIN* gene of the kiwifruit was used for normalization [53]. All analyses were repeated thrice using biological replicates.

### 4.6. Statistical Analysis

The PCA analysis and construction of the Venn diagram were performed using the package *prcomp* in the R software (www.r-project.org, 1 January 2020, the R Core Team, Auckland, New Zealand). Both HCA (hierarchical cluster analysis) and PCC were conducted using the R package *pheatmap*. For analyzing the differentially expressed metabolites, the metabolites with a fold change ≥1 and fold change ≤ 0.5 were selected, and alignment tests were performed to avoid overfitting. Finally, to perform pathway enrichment analysis, the KEGG (Kyoto Encyclopedia of Genes and Genomes) database (http://www.genome.Jp/kegg/, 1 January 2020) was used to find metabolic pathways that were highly enriched in differential metabolites between root and fruit. Hypergeometric tests were conducted to determine the differences between the root and fruit groups, and pathways were considered to be significantly different at *p* < 0.05 [54]. Relative expressions of genes were calculated using the 2^−∆∆Ct^ method [55], and the Origin 2021 software was used for plotting graphs. The IBM^®^ SPSS^®^ software (IBM, Washington, DC, USA) was used to determine statistically significant differences.

## Figures and Tables

**Table 1:** Statistical information of the identified metabolites.

Mode	All Metabolites	MS1 KEGG
POS	201	73
NEG	209	62
Total	410	135

“Mode” indicates that the mode of the MS analysis was mainly divided into a positive ion mode (POS) and a negative ion mode (NEG). “All metabolites” indicates the substances extracted by UPLC-MS/MS; “MS1 KEGG” indicates the metabolites that were annotated to the KEGG pathway.

**Table 2:** Metabolites that were significantly different between the root and flesh.

Metabolite Name	Comparison Group	Log_2_ FC	KEGG Pathway
Kaempferol-3-rhamnoside	DH_vs_R	2.39	Flavone and flavonol biosynthesis
	HT_vs_R	17.01	
	JY_vs_R	2.47	
	RZ_vs_R	6.18	
L-Epicatechin	DH_vs_R	3.84	Flavonoid biosynthesis
	HT_vs_R	19.68	
	JY_vs_R	4.55	
	RZ_vs_R	5.94	
Phloretin	DH_vs_R	13.10	Flavonoid biosynthesis
	HT_vs_R	13.10	
	JY_vs_R	13.10	
	RZ_vs_R	13.10	
Trifolin	DH_vs_R	5.53	Flavone and flavonol biosynthesis
	HT_vs_R	17.50	
	JY_vs_R	5.83	
	RZ_vs_R	3.25	

**Table 3:** The genes with significant differences between the root and flesh.

Groups	All DEGs		MS1 KEGG	
	Up	Down	Up	Down
DH_vs_R	12,191	9238	1817	2248
HT_vs_R	14,838	9624	2331	2349
JY_vs_R	11,895	9243	1788	2263
RZ_vs_R	15,814	8454	2573	2058

“Groups” indicates a comparison between two groups, taking A_vs_B as an example, A is regarded as the control group and B is the experimental group. “MS1 KEGG” indicates the DEGs that were annotated to the KEGG pathway.

**Table 4:** A summary of the functional description of 13 DEGs.

Gene_ID	Gene_Description	TF_Family
*Acc24966*	Chalcone synthase	Chal_sti_synt_N
*Acc18331*	Flavonoid-3’-hydroxylase	p450
*Acc11227*	Caffeoyl-CoA-O-methyltransferase	–
*Acc12443*	BAHD acyltransferase	–
*Acc23632*	Dihydroflavonol 4-reductase	Epimerase
*novel.5207*	Caffeoyl-CoA-O-methyltransferase	–
*novel.82*	Trans-cinnamate-4-monooxygenase	p450
*Acc00871*	Cytochrome P450 CYP73A100 like	p450
*Acc23312*	Caffeoyl-CoA-O-methyltransferase	–
*Acc32390*	Flavonoid-3’,5’-hydroxylase	p450
*Acc04926*	Ethylene-responsive transcription factor RAP2–4 like	AP2
*Acc25114*	Ethylene-responsive transcription factor 4 like	AP2
*Acc02924*	Transcription factor bHLH62 like	bHLH

**Table 5:** The correlation analysis of the four DEMs and 13 DEGs.

	Kaempferol-3-Rhamnoside	L-Epicatechin	Phloretin	Trifolin
*Acc24966*	0.878 **	0.838 **	NA	NA
*Acc18331*	0.812 **	NA	NA	NA
*Acc11227*	NA	NA	NA	NA
*Acc12443*	NA	NA	NA	NA
*Acc23632*	NA	NA	NA	NA
*novel.5207*	NA	NA	NA	NA
*novel.82*	NA	NA	1**	NA
*Acc00871*	0.82	NA	NA	NA
*Acc23312*	0.819 **	NA	NA	NA
*Acc32390*	NA	NA	NA	NA
*Acc04926*	0.848 **	0.909 **	NA	0.889 **
*Acc25114*	0.934 **	0.923 **	NA	NA
*Acc02924*	−0.849 **	NA	NA	NA

NA represents irrelevant; ** indicates a statistically significant correlation at *p* < 0.01.

## Data Availability

Not applicable.

## Supplementary Materials

The following are available online at https://www.mdpi.com/article/10.3390/ijms24021299/s1.
