# Supplementary material for: Comprehensive Analysis of Metabolome and Transcriptome in Fruits and Roots of Kiwifruit
Source: Int J Mol Sci. 2023 Jan 9;24(2):1299. doi: 10.3390/ijms24021299 (PMC9861564; doi:10.3390/ijms24021299)
Supplement: Supplementary file 1 [file ijms-24-01299-s001.zip › ijms-2111364-supplementary.pdf]

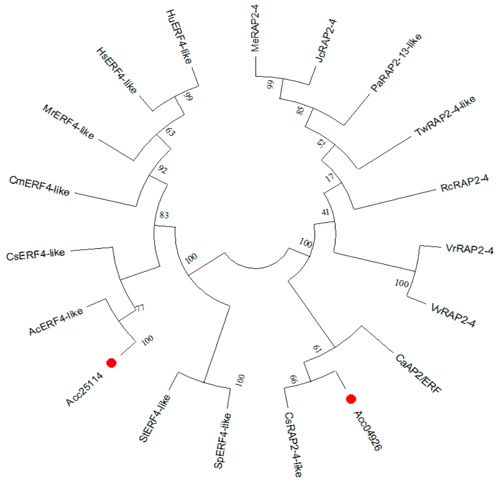

Figure S1. Evolutionary tree analysis of AP2 transcription factor. Ca: *Camptotheca acuminata,* Pa: *Populus alba,* Cs: *Camellia sinensis,* Vr: *Vitis riparia,* Me: *Manihot esculenta,* Jc: *Jatropha curcas,* Vv*: Vitis vinifera,* Rc: *Rosa chinensis,* Ac: *Actinidia chinensis var. Chinensis,* Sp: *Solanum pennellii,* St: *Solanum tuberosum,* Mr: *Morella rubra,* Cm: *Cucurbita maxima,* Hs: *Hibiscus syriacus,* Hu: *Herrania umbratica.*

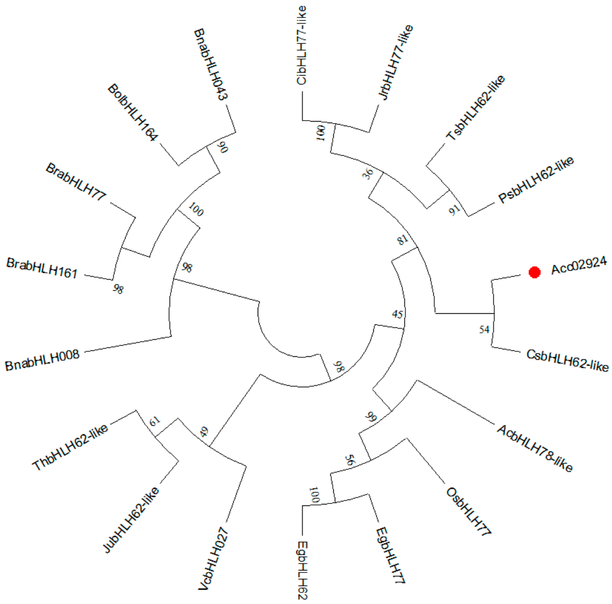

Figure S2. Evolutionary tree analysis of bHLH transcription factor. Cs: *Camellia sinensis,* Ju: *Juglans microcarpa* × *Juglans regia,* Ci: *Carya illinoinensis,* Jr: *Juglans regia,* Th: *Tarenaya hassleriana,* Vc: *Vaccinium corymbosum,* Bra: *Brassica rapa,* Bol: *Brassica oleracea,* Bn: *Brassica napus,* Ts: *Telopea speciosissima,* Ps: *Papaver somniferum,* Ac: *Ananas comosus,* Eg: *Elaeis guineensis,* Os: *Oryza sativa Japonica group.*

Table S1 Primer sequence

Gene name	Primer sequence (5' to 3')
β-ACTIN-F	TGAGAGATTCCGTTGCCCAGAAGT
β-ACTIN-R	TTCCTTACTCATGCGGTCTGCGAT
AcC4H-F	TCGAGTGGGGAATTGCAGAG
AcC4H-R	AGGTTCATGTGAGGCACGAG
AcCHS-F	ATCACAGCCGTTACCTTCCG
AcCHS-R	CTGGAAGGATGGTCTGAGCG
AcRAP2-4-F	GCGGCTTTGGCTACGATAAG
AcRAP2-4-R	TCCTCGTATGGCGAACTCTG
AcAP2-4-F	GGGGTAGGGGCTTGTTTAGG
AcAP2-4-R	GTTTCGTGGGAGGCGAATCT
AcbHLH62-F	TGTTTCCATGTCGCGGATCT
AcbHLH62-R	GCATCACCGTGAAAGTCGTG
AcPAL-F	TACATCGATGACCCGTGCAG
AcPAL-R	CCTGTTCGGAATTCCTCCCC
Ac4CL-F	ACGATCCAGAGGCCACAAAG
Ac4CL-R	ACCGATCGACGATGAAGAGC
AcHCR-F	AACGTCGACCTTGTCATCCC
AcHCR-R	TTCAAGCGACCAGCCATAGG
AcCHI-F	CAGTTGGAGACGGAACCCAA
AcCHI-R	TGAGGAATTCCCATCCGCAC
AcF3H-F	GTGGCGTACAACGTGTTCAG
AcF3H-R	ACACGCCTCCACGATCTTAC
AcFLS-F	TGTTGAACCTCACACCGACA
AcFLS-R	TGTGGAGGCACAACAAACAC
AcF3'H-F	CTCCACGGAAGGTTCGACTC
AcF3'H-R	TGCTATGGCCCACTCAACTG
AcDFR-F	GGCTATATTGTCCGCGCAAC
AcDFR-R	CACAGGGTCAGGTTCGTCTC
AcANS-F	TCTTCCACCTTGCTTACCCG
AcANS-R	CTTCGCGTACTCGCTTGTTG
AcANR-F	GCCTTGTCATGGACGAGAGT
AcANR-R	GAGCGTGGGAATGACCGTAA
AcCCO-F	AGGTGTGATCGGCTACGACA
AcCCO-R	TCCTAGGGTCCACAGCCAAA
